# Effects of Oral Liposomal Glutathione in Altering the Immune Responses Against *Mycobacterium tuberculosis* and the *Mycobacterium bovis* BCG Strain in Individuals With Type 2 Diabetes

**DOI:** 10.3389/fcimb.2021.657775

**Published:** 2021-06-03

**Authors:** Kimberly To, Ruoqiong Cao, Aram Yegiazaryan, James Owens, Timothy Nguyen, Kayvan Sasaninia, Charles Vaughn, Mohkam Singh, Edward Truong, Albert Medina, Edith Avitia, Jose Villegas, Christal Pham, Airani Sathananthan, Vishwanath Venketaraman

**Affiliations:** ^1^ Graduate College of Biomedical Sciences, Western University of Health Sciences, Pomona, CA, United States; ^2^ College of Osteopathic Medicine of the Pacific, Western University of Health Sciences, Pomona, CA, United States

**Keywords:** cytokine - immunological terms, type 2 diabetes, mycobacteria, oxidative stress, host directed therapy, immune responses

## Abstract

The World Health Organization (WHO) has identified type 2 diabetes (T2DM) as a neglected, important, and re-emerging risk factor for tuberculosis (TB), especially in low and middle-income countries where TB is endemic. In this clinical trial study, oral liposomal glutathione supplementation (L-GSH) or placebo was given to individuals with T2DM to investigate the therapeutic effects of L-GSH supplementation. We report that L-GSH supplementation for 3 months in people with T2DM was able to reduce the levels of oxidative stress in all blood components and prevent depletion of glutathione (GSH) in this population known to be GSH deficient. Additionally, L-GSH supplementation significantly reduced the burden of intracellular mycobacteria within *in vitro* granulomas generated from peripheral blood mononuclear cells (PBMCs) of T2DM subjects. L-GSH supplementation also increased the levels of Th1-associated cytokines, IFN-γ, TNF-α, and IL-2 and decreased levels of IL-6 and IL-10. In conclusion our studies indicate that oral L-GSH supplementation in individuals with T2DM for three months was able to maintain the levels of GSH, reduce oxidative stress, and diminish mycobacterial burden within *in vitro* generated granulomas of diabetics. L-GSH supplementation for 3 months in diabetics was also able to modulate the levels of various cytokines.

## Introduction

Tuberculosis (TB) caused by *Mycobacterium tuberculosis* (*M. tb*) continues to be one of the leading causes of death from a single infectious agent in humans (ranked above HIV/AIDS) and is one of the top 10 causes of death worldwide (Geneva: World Health Organization, 2019). From the estimated 1.7 billion people infected with *M. tb*, only a small portion of about 5-10% develop the disease in their lifetime (Geneva: World Health Organization, 2019). Tuberculosis remains a public health threat despite an average decline in TB incidence because of the rise in comorbidities involving conditions such as diabetes and HIV. Several studies, including a systematic review of 13 observational studies, showed that T2DM was associated with increased TB risk, regardless of the study design or population (Jeon and Murray, 2008). Individuals with T2DM, characterized by hyperglycemia, show an increase in ROS, proinflammatory cytokines, and an altered GSH synthesis and metabolism. Several studies have confirmed that GSH concentration is compromised in erythrocytes and plasma of people with diabetes along with the decreased levels of enzymes that participate in GSH biosynthesis (Yoshida et al., 1995; Lagman et al., 2015). Intracellular redox homeostasis is characterized by the ratio of oxidized glutathione (GSSG) to reduced glutathione (GSH), with reduced GSH being the active antioxidant. On the other hand, GSSG is non-functional and quickly converted back to GSH by glutathione reductase (GSR). Under non-pathological conditions, the ratio of GSSG to GSH is 1%. The depletion of GSH, combined with ROS overproduction in people with diabetes, has resulted in an altered immunity that has contributed to the increasing prevalence of TB-T2DM co-infection and poorer outcomes for people with diabetes (Restrepo, 2016). The probability of developing an active TB disease increases with risk factors such as HIV, diabetes, undernutrition, smoking, and alcohol consumption (Geneva: World Health Organization, 2019). People living with HIV are at 19 times higher risk for developing active TB than the general global population, while individuals with T2DM have three times the risk (Restrepo, 2016).

The standard therapeutic regimen to treat active TB recommended by the Center for Disease Control and Prevention (CDC) is a multidrug regimen of antibiotics taken from 6 to 9 months known as DOTS (Directly Observed Treatment, Short-course) (Centers for Disease Control and Preventation, 2016). DOTS comprise of four antibiotics: isoniazid (INH), rifampicin (RIF), pyrazinamide (PZA), and ethambutol (ETH) for two months (initial phase) followed by INH, and RIF for four months (continuation phase) (Lagman et al., 2015). There are many adverse effects, ranging from serious ones like hepatotoxicity to minor ones like peripheral neuropathy, joint, pain, visual impairment, nausea, and vomiting (El-Sadr et al., 2001; Ramappa and Aithal, 2013; Sharma et al., 2013). Due to the lengthy duration of treatment and adverse side effects, there is a high drop-out rate from DOTS therapy. Thus, non-compliance is directly contributing to the development of multidrug and extensively drug resistance (MDR/XDR)-TB. Multidrug-resistant and extensively drug-resistant TB are already emerging worldwide and has become a major public health threat (Singh and Subbian, 2018).

One approach to combat *M*. *tb* infection is to identify host-directed therapies (HDT) or strategies that can modulate specific host immune pathways for more effective killing of *M*. *tb.* HDT for *M*. *tb* aims to augment an individuals’ immune response or metabolism to optimize the use of antibiotics for better clearance of *M*. *tb* (Singh and Subbian, 2018).

A potential adjunct therapy is glutathione (GSH). GSH is a tripeptide synthesized in the cell cytosol from the amino acids precursors: glutamate, cysteine, and glycine, by two ATP-requiring enzymes: glutamate-cysteine ligase (GCL) and GSH synthetase (GSS). First, glutamate is coupled to cysteine by GCL. Second, the γ-glutamylcysteine product is coupled to glycine *via* GSH synthetase (GSS) to produce GSH or reduced GSH (Teskey et al., 2018a). GSH has an active thiol group present on the cysteine residue and functions by directly detoxifying reactive oxygen species (ROS) and reactive nitrogen species (RNS) or indirectly *via* GSH-dependent peroxidase-catalyzed reactions (Cooper et al., 2011). GSH is also required for other critical cell processes, including cell differentiation, proliferation, and apoptosis (Ballatori et al., 2009).

T2DM is a chronic metabolic disease characterized by hyperglycemia, prolonged elevated levels of glucose, and insulin resistance. T2DM and hyperglycemia are also associated with oxidative stress from the overproduction of reactive oxygen species (ROS) and decreased efficiency of scavengers systems (Collier et al., 1990; Powell et al., 2004). Oxidative stress and hyperglycemia have been shown to alter GSH synthesis and metabolism in cases of diabetes (Dave and Kalia, 2007).

Furthermore, our lab has previously shown that individuals with T2DM have lowered levels of GSH due to decreased levels of both GCL and GSS protein compared to healthy subjects (Lagman et al., 2015). In the same study, increased levels of transforming growth factor-beta (TGF-β), a cytokine known to reduce the expression of GCL, were observed in plasma samples from T2DM subjects when compared to the healthy group. These lowered levels of GSH and the GSH metabolism enzymes in T2DM individuals led to increased susceptibility to an *M*. *tb* infection (Lagman et al., 2015). Our lab has also shown that GSH deficiency is correlated with increased levels of free radicals and intracellular *M*. *tb* viability (Venketaraman et al., 2008). Additionally, our lab has found that GSH has direct antimycobacterial activity, thus functioning as an effector molecule in the innate defense against *M*. *tb* (Venketaraman et al., 2005). *In vitro* restoration of GSH with the glutathione precursor: N-Acetyl Cysteine (NAC), combined with antibiotics, INH and RIF, resulted in bolstering cytokine modulation and complete clearance of *M*. *tb* infection in granulomas generated from PBMCs from healthy subjects (Teskey et al., 2018b). Moreover, *in vitro* GSH supplementation in macrophages isolated from individuals with T2DM resulted in improved control of *M*. *tb* infection (Teskey et al., 2018b). We therefore determined if oral supplementation with liposomal glutathione (L-GSH) for three months (12 weeks) in individuals with T2DM will reduce oxidative stress and enhance antimycobacterial responses against *in vitro M. tb* or *M. bovis* BCG infections, alongside antibiotics.

## Material and Methods

### Study Design and Intervention

**Figure 1:**
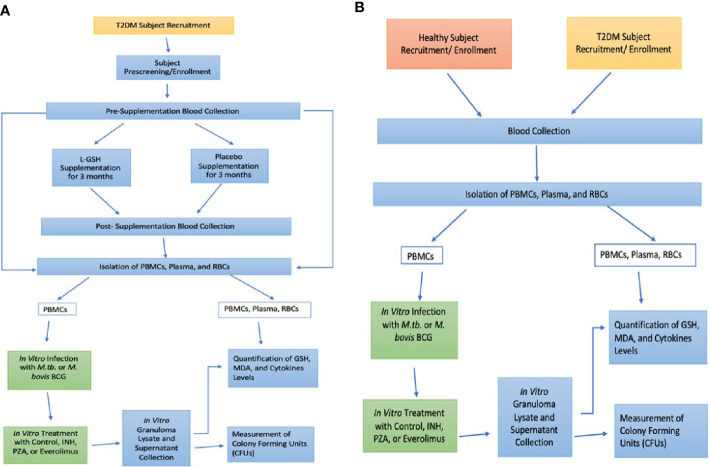
Study Design. L-GSH Clinical Trial Study design in subjects with T2DM **(A)** and Preclinical Study Design in Healthy and T2DM Subjects **(B)**.

This study was a randomized, double-blinded, placebo-controlled, parallel-group clinical trial conducted at the Western University of Health Sciences. This study was approved by Western University of Health Sciences Institutional Review Board (IRB); protocol #FB17/IRB/031. All participants gave written informed consent before enrollment into the study. The experimental group received oral liposomal glutathione (L-GSH) for three months and while the placebo group received the oral empty liposomes for three months. Each subject in both groups took 1.5 teaspoons of oral liposomal glutathione or empty liposomes twice a day (morning and evening), for a total of 3 teaspoons daily (15 ml or 1260 mg of L-GSH). Your Energy Systems manufactured the oral L-GSH supplement as ReadiSorb™ LRG, a dietary supplement supplying reduced form of GSH in liposomes suspended in liquid. The same company manufactured the placebo treatment with the same materials, but with empty liposomes. All the components in ReadiSorb™ LRG are natural and considered GRAS (Generally Recognized as Safe) by the FDA. No toxicity in children using LRG daily for two months was documented (Kern et al., 2011). Previous studies also documented no toxicity in healthy and HIV-positive subjects using the same LRG daily for three months with the same dose in this study (Ly et al., 2015; Valdivia et al., 2017). Your Energy Systems has patented this ReadiSorb™ LRG as an oral liposomal glutathione supplement. **Figure 1A** shows an overview of the entire study design for the L-GSH clinical study with T2DM subjects.

### Subject Selection, Clinical Encounters, and Blood Collection

Patients were recruited from the on-campus clinic, the Patient Care Center (PCC) of Western University of Health Sciences, from October 2018 until the target sample size was reached for both the experimental and placebo-controlled arm. Enrolled subjects were between the ages of 18-65 years old, positive for T2DM with glycated hemoglobin (HbA1c) levels between 7-13%. Subjects enrolled were also negative for HIV and had no history of latent tuberculosis infection or liver function abnormalities. Past infections with hepatitis and frequent alcohol abuse could impair liver function and independently cause depletion of GSH levels (Teskey et al., 2018b). Antibodies against HIV were tested in all groups to confirm that all participants were negative for HIV through a pre-screening that included a comprehensive metabolic panel, HbA1c levels, hepatitis B surface antigen, and pregnancy test (if applicable). Exclusion criteria included an HIV-positive test result or abnormal liver function due to chronic viral hepatitis or alcohol abuse. The AUDIT questionnaire (Alcohol Use Disorders Identification Test) was given to determine if there was alcohol abuse. AUDIT questionnaire scores >8 indicated hazardous alcohol use and excluded from the study (Saunders et al., 1993). The exclusion criteria enabled enrollment of participants without liver abnormalities to provide a better association between T2DM, decreased GSH levels, and increased susceptibility to mycobacterial infections. During the pre-screening appointment, the clinician also took vital measurements, performed a physical exam, symptom, and medication review. After this initial pre-screening visit and cleared for enrollment, each subject met with the same clinician for a total of three more in-person visits: Visit #1 (Baseline), Visit #2 (Midway point), Visit #3 (Post-Treatment). 50 ml of venous blood was drawn and collected from subjects during the Pre-screening, Visit #1, and Visit #3, but not during the Visit #2 (midway-point). The blood collected was used for *in vitro* research studies in BSL-3 and BSL-2 facilities. Study subjects received $100 in cash for their participation in four installments of $25 during each clinical encounter: pre-screening, baseline (V1), midway point (V2), and post-treatment (V3). Eight healthy volunteers also made a one-time donation of their blood for preclinical studies to establish a baseline against the clinical trial T2DM subjects. **Figure 1B** shows an overview of the preclinical study design with healthy and T2DM subjects. Healthy volunteers gave written informed consent and were compensated with $10 in cash.

### Isolation and Storage of Peripheral Blood Mononuclear Cells, Plasma, and Red Blood Cells

Peripheral Blood Mononuclear Cells (PBMCs), plasma, and red blood cells (RBCs) were isolated from whole blood by density gradient centrifugation using ficoll histopaque (Sigma, St. Louis, MO, USA). Whole blood was layered on to ficoll histopaque in a 1:1 ratio and centrifuged at 25°C for 30 minutes at 1800 RPM with the slow brake to prevent disturbance of the gradient. Plasma and RBCs were aliquoted into separate vials and stored at -20°C. PBMCs were purified from other blood components and the ficoll histopaque by washing twice with 1x phosphate-buffered saline (PBS). PBMCs were then resuspended in 1ml of cold FBS for cryopreservation. FBS 20% DMSO was slowly added dropwise to the cell suspension for a final concentration of FBS 10% DMSO. This final suspension was aliquoted into 1ml vials and stored at -20°C for 1 hour and then stored to -80°C until used for *in vitro granulomas formation and* infection studies.

### Thawing Cryopreserved PBMCs

The vials of cryopreserved PBMCs in FBS 10% DMSO O were transferred from -80°C storage on dry ice to a warm water bath heated to 37°C. When only one ice crystal remained, the PBMCs were transferred to a new tube to be washed 2x with warm RPMI (Sigma, St. Louis, MO, USA) with 5% human AB serum (Sigma, St Louis, MO, USA) and centrifuged at 1200 RPM for 20 mins at 25°C. PBMCs were again resuspended in warm RPMI with 5% human AB serum, and 2mL of PBMCs was aliquoted and stored for future assays while the rest was distributed at 6 x 10^5^ cells/well onto 24-well plates (Corning, NY, USA) coated with.001% poly-lysine (Sigma, St. Louis, MO, USA). PBMC cell counts were determined with trypsin blue exclusion staining. Before *in vitro* infection, PBMCs were rested overnight in the 24-well plate in a cell incubator at 37°C with 5% CO_2_.

### *In Vitro* PBMC Infection and Treatment

Following the PBMCs resting overnight, they were infected with either the Erdman strain of *M. tb* or *M. bovis* BCG (BCG) at a multiplicity of infection (MOI) of 0.1:1 cell ratio. On the same day of infection, PBMCs were either sham treated (untreated) or treated with 1/10 the minimum inhibitory concentration 1/10 MIC of PZA (5 micrograms/ml), and (MIC) of INH (0.125 micrograms/ml). Infected and treated PBMCs were maintained at 37°C, with 5% CO_2_ until they were terminated at 8- or 15-days post-infection.

### Terminating *In Vitro* Granulomas to Determine Survival of *M. tb* or BCG

Infected *In vitro* generated granulomas were harvested at 8 and 15 days, post-infection to determine the survival *of M. tb* or BCG. Cell-free supernatants were collected and stored at -20°C for the future assays and granulomas were harvested by adding ice-cold sterile 1x PBS to wells, followed by gentle scraping to achieve maximum recovery of granuloma lysates and stored at -20°C. To prepare them for plating, granulomas lysates were vortexed, followed by a freeze/thaw cycle to rupture the cells to release intracellular *M. tb or* BCG. Cell supernatants and lysates were plated on 7H11 agar medium (Hi Media, Santa Maria, CA, USA) enriched with ADC (GEMINI, USA) and incubated at 37°C for three weeks to evaluate mycobacterial growth or survival under treatment conditions by counting the colony-forming units (CFU’s). CFU numbers were corrected for if any dilutions were made to the supernatant or lysates before plating.

### Quantifying Levels of Glutathione (GSH)

Levels of GSH were measured in all blood components (plasma, RBCs, uninfected PBMCs lysates) as well as in the *in vitro* granuloma lysates harvested at 8-days and 15-days post-infection. The *in vitro* granulomas were prepared from the PBMCs isolated from blood drawn from individuals with T2DM at baseline and three months post- supplementation for both the experimental and placebo study groups. We measured GSH concentrations by using the GSH Colorimetric Detection Kit from Invitrogen (Cat # EIAGSHC) following the manufacturer’s protocol. The rGSH (reduced GSH) was obtained by subtracting GSSG (oxidized glutathione) from the total GSH. All measurements were normalized by the total protein levels and the results reported in moles of GSH per gram of protein.

### Quantifying Levels of Malondialdehyde (MDA)

MDA levels were measured in all blood components (plasma, RBCs, uninfected PBMCs lysates) as well as in the *in vitro* granuloma lysates harvested at 8-days and 15-days post-infection. PBMCs isolated from individuals with T2DM at baseline and three months post-supplementation with either L-GSH or empty liposomes were used for generating *in vitro* granulomas. at. MDA was measured using the Thiobarbituric Acid Reactive Substances (TBARS) assay kit from Cayman Chemicals (Item No.10009055) following the manufacturer’s protocol. The assay allows for MDA to be measured from an MDA-TBA adduct forming from the reaction of MDA and TBARS under high temperature (90-100°C) and acidic conditions. This MDA-TBA adduct is then measured colorimetrically at 530-540 nm. All measurements were normalized by the total protein levels.

### Cytokine Measurements

Cytokines Levels of IFN-γ, TNF-α, IL-6, and IL-10, were measured in the plasma, uninfected PBMC lysates at pre-supplementation and post supplementation in both placebo and experimental group. Cytokine levels were also measured in the mycobacteria-infected *in vitro* granuloma supernatants to determine the effects of *in vitro* PZA + *in vivo* GSH treatments and *in vitro* INH + *in vivo* GSH treatments in altering the levels of these cytokines. Cytokine levels were measured using enzyme-linked immunosorbent (ELISA) assay kits: Human 1L-10 ELISA Ready-SET-Go from Affymetrix (Cat# 88-7106) and Human TNF-α Uncoated ELISA (Cat # 88-7346), Human IFN-γ Uncoated ELISA (Cat # 88-7316), and Human IL-6 Uncoated ELISA (Cat # 88-7066) from Invitrogen to quantity detection of cytokines. Assays were performed following the manufacturer’s protocol for each kit.

### Statistical Analysis and Sample Size

Statistical analysis was performed using GraphPad Prism Software 8 using one-way ANOVA for comparing more than two categories with Tukey corrections. When comparing two categories, the unpaired T-test was used with Welch corrections. Reported values are the mean values of each respective category. A p<0.05 was considered significant, indicated by an asterisk (*), hashmark (#), or the dollar sign ($). Two symbols (**, ##, or $$) denote significant difference below 0.005, three symbols (***, ###, or $$$) denote significant below 0.0001. An asterisk (*, **, ***) above a certain category indicates significance in comparison to the category directly to the left of it. A hash mark (#, ##, ###) above a certain category indicates significance in comparison between that category and another category that is two columns to the left of it. A dollar sign ($, $$, $$$) above a certain category indicates significance in comparison between that category and another category that is three columns to the left of it. The sample size of the clinical trial was eleven T2DM positive subjects in the L-GSH supplementation arm and seven T2DM positive subjects in the placebo supplementation arm. For the preclinical studies, the sample size was eight healthy volunteers and eleven T2DM positive subjects.

## Results

### Preclinical Results: Intracellular Survival of *M. tb* and BCG Within *In Vitro* Granulomas of Healthy Subjects Compared to T2DM Subjects

The main host defense mechanism against mycobacterial infection involves granuloma formation to encapsulate and limit the pathogen’s infection. Granulomas are formed by various immune cells, including macrophages, dendritic cells, T cells, fibroblasts, epithelioid histiocytes, giant cells, and natural killer cells (North and Jung, 2004). Previous research has shown that granuloma formation occurs approximately seven days post-PBMC infection with *M. tb* (Kapoor et al., 2013).

**Figure 2:**
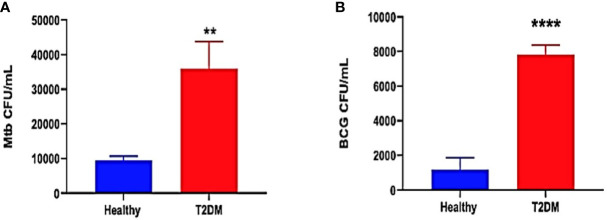
Preclinical Findings. Survival of *M. tb* **(A)** and BCG **(B)** in untreated *in vitro* granulomas generated from PBMCs isolated from healthy subjects and individuals with type 2 diabetes. PBMCs isolated from healthy subjects and individuals with type 2 diabetes were infected with Erdman strain of M*. tb* Granulomas were terminated at 8-days post-infection. Cell-free supernatants were collected and stored. Granulomas were lysed with ice-cold PBS. Supernatants and granuloma lysates were plated on 7H11 agar plates containing ADC to determine the survival of *M. tb* and BCG. (**) indicates a *p* <.005, (****) indicates *p*<0.0001.

The ability of immune cells derived from healthy and T2DM individuals to contain an *in vitro* mycobacterial infection was measured by determining the intracellular survival of *M. tb* and BCG within *in vitro* granulomas generated from PBMCs isolated from healthy and T2DM subjects. To confirm previous study findings between healthy and T2DM subjects, no treatment was added to the *in vitro* granulomas from the two study groups. In comparison to healthy subjects, there was a 3.5-fold increase in the burden of *M. tb* inside the granulomas from T2DM subjects (**Figure 2A**). Furthermore, there was a significant 8-fold increase in the growth of BCG inside granulomas from T2DM subjects compared to the healthy subjects (**Figure 2B**). Overall, T2DM subjects had a significant increase in the growth of BCG and *M. tb* within *in vitro* granulomas when compared to healthy individuals, thus confirming previous studies demonstrating T2DM individuals have a reduced ability to control the mycobacterial infections compared to healthy individuals (**Figures 2A, B**).

### Preclinical Results: Quantification of GSH, MDA, IL-6, TNF-α, and IFN-γ Levels in Healthy Compared to T2DM Subjects

**Figure 3:**
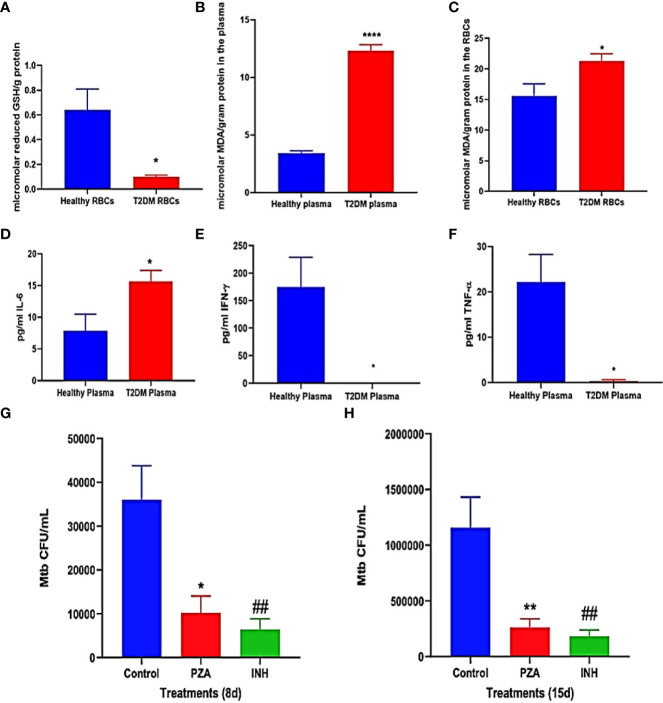
**(A–E)** Preclinical Findings. Quantification of reduced GSH (GSH), malondialdehyde (MDA), IL-6, IFN-γ, and TNF-α in healthy subjects and individuals with T2DM. Levels of GSH in the RBCs **(A)** were determined by spectrophotometry using an assay kit from Invitrogen. Levels of malondialdehyde in the plasma **(B)** and RBCs **(C)** were measured by spectrophotometry using an assay kit from Invitrogen. IL-6 **(D)**, IFN-γ **(E)**, and TNF-α **(F)** levels in the plasma samples isolated from healthy subjects and individuals with type 2 diabetes were determined by ELISA (Invitrogen). **(G, H)** Survival of M. tb in untreated, PZA-treated, and INH-treated in vitro granulomas generated from PBMCs isolated from individuals with type 2 diabetes. PBMCs isolated from individuals with T2DM were infected in vitro with Erdman strain of M. tb and were either untreated (control), treated in vitro with PZA (5 mg/ml), or INH (0.0125 mg/ml). Granulomas were terminated at 8-days **(G)** and 15-days **(H)** post-infection. Cell-free supernatants were collected and stored. Granulomas were lysed with ice-cold PBS. Supernatants and granuloma lysates were plated on 7H11 agar plates containing ADC to determine the survival of M. tb. A* p*<0.05 is considered significant indicated by an asterisk (*). (**) indicates a *p* <.005, (****) indicates *p*<0.0001, (##) indicates a *p* <.005.

For confirmation and demonstration of previous study findings, the levels of the reduced form of GSH, MDA, IL-6, TNF-α, and IFN-γ were compared in healthy and T2DM groups and were measured in the plasma and RBCs isolated from peripheral blood of healthy and T2DM subjects. Malondialdehyde (MDA) is a byproduct of lipid peroxidation and serves as an important marker for oxidative stress in cells and tissues (Armstrong and Hart, 1971; Yagi, 1994). The interactions between mycobacteria and granulomatous cells of the innate and the adaptive immunity result in the secretions of cytokines, notably IFN-γ, TNF-α, IL-6, and IL-10. These cytokines regulate immune responses against mycobacterial infection (O’Garra and Britton, 2017). Compared to healthy subjects, T2DM subjects had a significant decrease of more than 4-fold in the levels of the reduced form of GSH in the RBCs (**Figure 3A**). Decreased levels of the reduced form of GSH in T2DM correlated with a significant increase in the levels of MDA in the plasma (**Figure 3B**) and RBCs (**Figure 3C**) from T2DM subjects when compared to healthy subjects. There was also a significant increase in the levels of IL-6 in the plasma samples of T2DM subjects compared to healthy subjects (**Figure 3D**). T2DM subjects also had decreased levels of IFN-γ (**Figure 3E**) and TNF-α (**Figure 3F**) in the plasma samples when compared to healthy subjects.

Overall, individuals in the T2DM group had significantly decreased levels of the reduced form of GSH and increased levels of the oxidative stress marker MDA, compared to the healthy group (**Figures 3A–C**). Additionally, compared to the healthy group, the T2DM group has significantly increased levels of the proinflammatory cytokine IL-6 and decreased levels of IFN-γ (**Figure 3E**) and TNF-α (**Figure 3F**) in the plasma samples. These results reaffirm previous study conclusions about GSH, MDA, and cytokine levels between healthy and T2DM individuals (Lagman et al., 2015).

### Preclinical Results: Intracellular Survival of *M. tb* Within Antibiotic-Treated *In Vitro* Granulomas of T2DM Subjects

Intracellular survival of *M. tb* was determined within *in vitro* granulomas of infected-PBMCs from T2DM subjects that were either untreated (control), treated *in vitro* with PZA (one-time addition at 5 μg/ml) or with INH (one-time addition at 0.0125 μg/ml). This measurement was used to investigate the efficacy of using 1/10 of the MIC of PZA and INH in controlling *M. tb* infection within *in vitro* granulomas inT2DM group, with the basis coming from a previous finding in our lab that showed MIC of PZA and INH was able to reduce *M. tb*. burden in healthy individuals (Abrahem et al., 2019). However the efficacy of these antibiotics at 1/10 MIC against *M.tb* in individuals with T2DM was not previously determined. When compared to the control group, PZA treatment resulted in more than a 60% reduction in the viability of *M. tb* within *in vitro* granulomas harvested at both 8 days (**Figure 3F**) and 15 days post-infection (**Figure 3G**). There was also a decrease in the viability of *M. tb* observed with INH treatment, compared to the control group at both 8 days (**Figure 3F**) and 15 days post-infection (**Figure 3G**). Overall, the treatment of *in vitro* granulomas generated from PBMCs isolated from T2DM subjects with 1/10 MIC for PZA (5 μg/ml) and 1/10 for MIC INH (0.0125 μg/ml) treatment resulted in a significant decrease in the *M. tb* burden (**Figures 3F, G**).

### Clinical Trial Findings: Quantification of IL-6, MDA, and GSH Levels in T2DM Subjects

**Figure 4:**
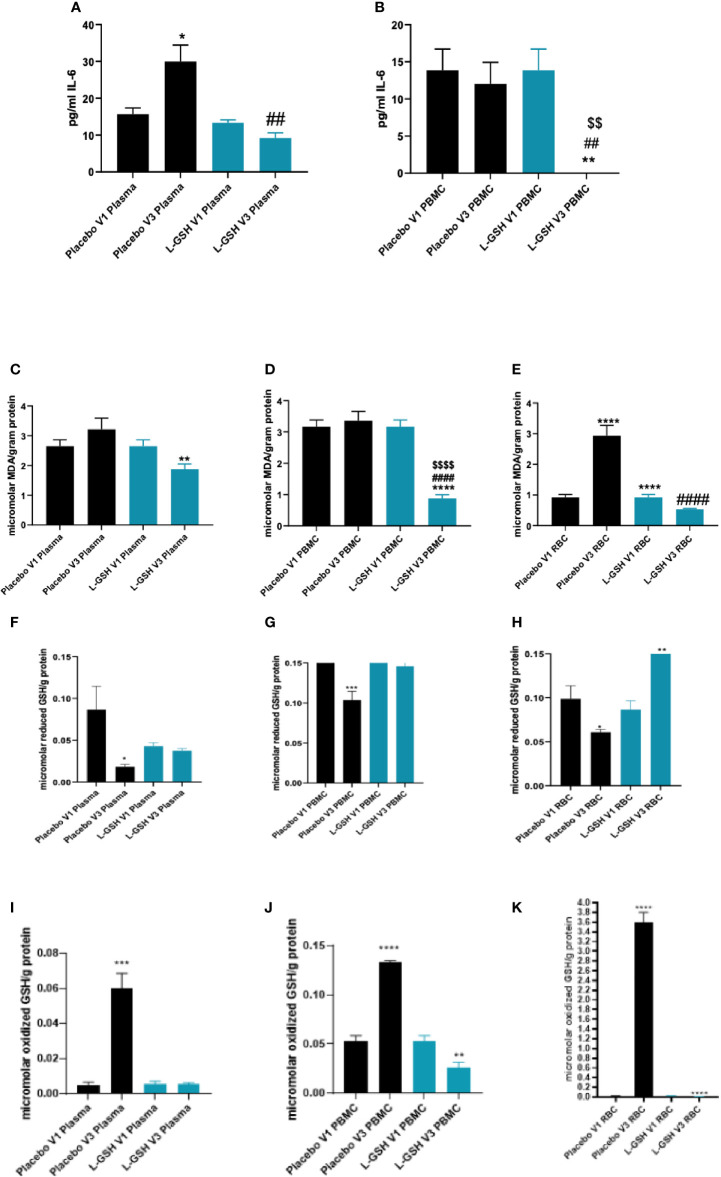
Clinical trial findings. Quantification of IL-6, MDA and GSH in blood samples from individuals with T2DM on L-GSH/or placebo supplement. Levels of IL-6 in the plasma **(A)** and PBMCs **(B)** isolated from individuals with T2DM at pre- and post- L-GSH/or placebo supplementation were determined by spectrophotometry using an assay kit from Invitrogen. V1 represents plasma samples **(A)** or PBMCs **(B)** isolated from subjects with T2DM prior to L-GSH/or placebo supplementation. V3 represents plasma samples **(A)** or PBMCs **(B)** isolated from subjects with T2DM at three months post- L-GSH/or placebo supplementation. Levels of malondialdehyde in the PBMCs **(C)**, plasma **(D)**, and RBCs **(E)** isolated from individuals with T2DM at pre- and post- L-GSH/or placebo supplementation were determined by spectrophotometry using an assay kit from Invitrogen. V1 represents plasma **(C)**, PBMCs samples **(D)**, or RBCs **(E)** isolated from subjects with T2DM prior to L-GSH/or placebo supplementation. V3 represents plasma **(C)**, PBMCs samples **(D)**, or RBCs **(E)** isolated from subjects with T2DM at three months post- L-GSH/or placebo supplementation. V1 represents samples isolated from subjects with T2DM prior to L-GSH/or placebo supplementation. V3 represents samples isolated from subjects with T2DM at three months post- L-GSH/or placebo treatment. Levels of reduced form of GSH **(F–H)**) and oxidized form of GSH (GSSG) **(I–K)** in the plasma **(F, I)**, PBMCs **(G, J)**, and RBCs samples **(H, K)** isolated from individuals with T2DM at pre- and post- L-GSH/or placebo supplementation were determined by spectrophotometry using an assay kit from Invitrogen. V1 represents samples isolated from subjects with T2DM prior to L-GSH/or placebo supplementation. V3 represents samples isolated from subjects with T2DM at three months post- L-GSH/or placebo treatment. A *p*<0.05 is considered significant indicated by an asterisk (*), (**) indicates a *p* <.005, (***) indicates *p*<0.0005, (****) indicates *p*<0.0001, (##) indicates a *p* <.005, (####) indicates *p*<0.0001, ($$) indicates a *p* <.005, ($$$$) indicates *p*<0.0001.

Levels of IL-6 were quantified in plasma and PBMCs isolated from T2DM subjects at pre- and post- L-GSH/or placebo supplementation. L-GSH supplementation in T2DM subjects for 3-months resulted in a decrease in the levels of IL-6 in both the plasma (**Figure 4A**) and PBMCs (**Figure 4B**). Alternatively, in the group that took 3-months of supplementation with placebo, there was an observed significant increase in levels of IL-6 in the plasma (**Figure 4A**), and no significant differences observed in IL-6 levels within PBMCs (**Figure 4B**). Overall, L-GSH supplementation for 3-months in individuals with T2DM decreased levels of the proinflammatory cytokine IL-6, while the placebo group had a significant increase in IL-6 levels.

MDA, a measure of oxidative stress, was quantified in the plasma, PBMCs, and RBCs isolated from T2DM subjects at pre- and post- L-GSH/or placebo supplementation. L-GSH supplementation for 3-months resulted in a significant decrease in the levels of MDA in plasma (**Figure 4C**), PBMCs (**Figure 4D**), and RBCs (**Figure 4E**). In the group that took the placebo supplement for 3 months, we observed no significant differences in the levels of MDA in plasma (**Figure 4C**) and PBMCs (**Figure 4D**). Importantly, the group that took the placebo was observed to have a significant increase in MDA levels in RBCs (**Figure 4E**). Overall, L-GSH supplementation for 3-months resulted in reduced MDA levels, while the placebo group was observed to have a significant increase in the levels of MDA in the RBCs and no significant change in the levels of MDA in the plasma and PBMCs.

Levels of reduced and oxidized forms of GSH (GSSG) were also quantified in plasma, PBMCs, and RBCs isolated from T2DM subjects at pre- and post- L-GSH/or placebo supplementation. When compared to before placebo supplementation, post-placebo supplementation in individuals with T2DM resulted in a significant decrease in the levels of reduced form of GSH in plasma (**Figure 4F**), in PBMCs (**Figure 4G**), and in RBCs (**Figure 4H**) samples. For the L-GSH supplementation group, the levels of reduced form of GSH remained relatively the same and were not significantly different between pre- and post- L-GSH supplementation in plasma (**Figure 4F**) and PBMCs (**Figure 4G**). However, for the L-GSH group, there was a significant increase in the levels of reduced form of GSH in the RBCs at 3-months post L-GSH supplementation(**Figure 4H**). On the other hand, there was a significant increase in the levels of oxidized GSH (GSSG) in the plasma (**Figure 4I**), PBMCs (**Figure 4J**) and RBCs (**Figure 4K**) samples from individuals with T2DM at 3-months post-placebo supplementation. For the L-GSH supplementation group, there was a significant decrease in the levels of oxidized GSH in PBMCs (**Figure 4J**) and RBCs (**Figure 4K**), but not in plasma (**Figure 4I**).

Overall, these results show that a 3-month clinical intervention with L-GSH supplementation in T2DM subjects resulted in significantly decreased levels of the proinflammatory cytokine IL-6, and a decrease in the oxidative stress marker MDA. Supplementation with placebo for 3-months had no observed significant changes or the opposite change of L-GSH supplementation for IL-6 and MDA. L-GSH supplementation for three months was also able to significantly decrease the levels of GSSG in PBMCs and RBCs while significantly increasing the levels of reduced form of GSH in RBCs and maintaining levels of reduced form of GSH in plasma and PBMCs. Alternatively, the placebo group had increased levels of GSSG and decreased levels of reduced form of GSH post- placebo supplementation.

### Clinical Trial Findings: Quantification of Cytokines IFN-γ, TNF-α, IL-2, and IL-10 Levels in Plasma and PBMCs From T2DM Subjects

Levels of cytokines IFN-γ, TNF-α, IL-2, and IL-10 were quantified in plasma and PBMCs isolated from T2DM subjects at pre- and post- L-GSH/or placebo supplementation. PBMC lysate was utilized in our experimentation to find intracellular cytokine levels. Though this method is not typically used to measure cytokine levels as compared to the more common plasma measurements, it can be an important method of measure due to the short half-life of cytokines and their localization in production, thus many studies have utilized this alternate method (Sullivan et al., 2000; Cloez-Tayarani, 2003; Jensen et al., 2007; Stoffels et al., 2015).

**Figure 5:**
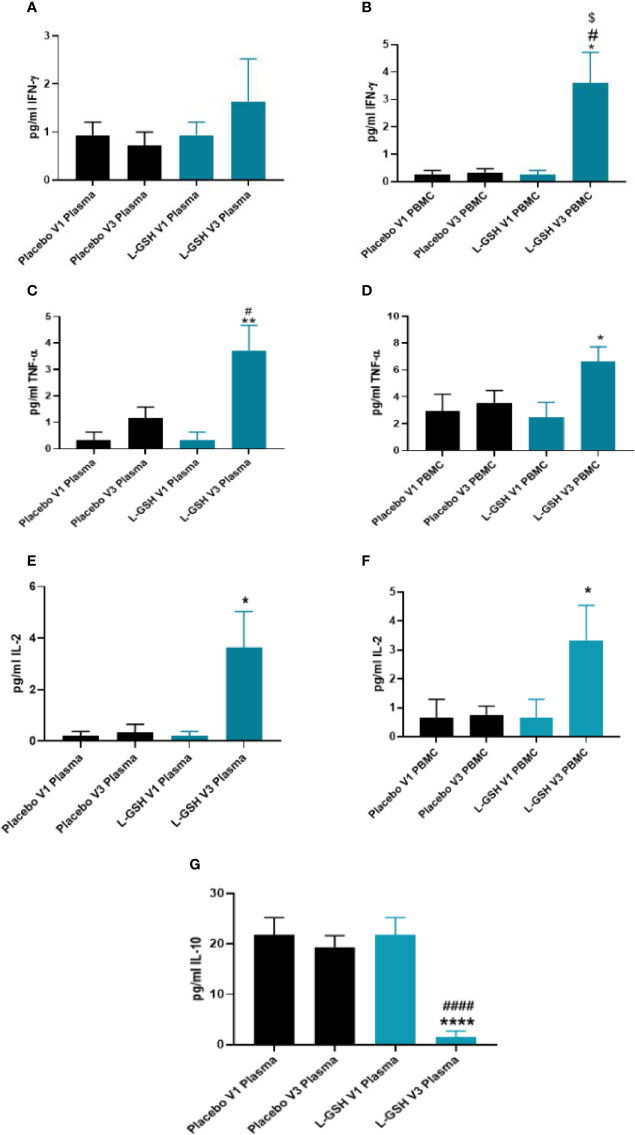
Clinical trial findings. Quantification of cytokines in plasma samples and PBMCs of individuals with T2DM on L-GSH/or placebo supplement. Levels of IFN-γ in the plasma **(A)** and PBMCs **(B)** isolated from individuals with T2DM at pre- and post- L-GSH/or placebo supplementation were determined by spectrophotometry using an assay kit from Invitrogen. V1 represents plasma samples **(A)** or PBMCs **(B)** isolated from subjects with T2DM prior to L-GSH/or placebo supplementation. V3 represents plasma samples **(A)** or PBMCs **(B)** isolated from subjects with T2DM at three months post- L-GSH/or placebo supplementation. Levels of TNF-α in the plasma **(C)** and PBMCs **(D)** isolated from individuals with T2DM at pre- and post- L-GSH/or placebo supplementation were determined by spectrophotometry using an assay kit from Invitrogen. V1 represents plasma samples **(C)** or PBMCs **(D)** isolated from subjects with T2DM prior to L-GSH/or placebo supplementation. V3 represents plasma samples **(C)** or PBMCs **(D)** isolated from subjects with T2DM at three months post- L-GSH/or placebo supplementation. Levels of IL-2 in the plasma **(E)** and PBMCs **(F)** isolated from individuals with T2DM at pre- and post- L-GSH/or placebo supplementation were determined by spectrophotometry using an assay kit from Invitrogen. V1 represents plasma samples **(E)** or PBMCs **(F)** isolated from subjects with T2DM prior to L-GSH/or placebo supplementation. V3 represents plasma samples **(E)** or PBMCs **(F** isolated from subjects with T2DM at three months post- L-GSH/or placebo supplementation. Levels of IL-10 in the plasma **(G)** isolated from individuals with T2DM at pre- and post- L-GSH/or placebo supplementation were determined by spectrophotometry using an assay kit from Invitrogen. V1 represents plasma samples isolated from subjects with T2DM prior to L-GSH/or placebo supplementation. V3 represents plasma samples isolated from subjects with T2DM at three months post- L-GSH/or placebo supplementation. A *p*<0.05 is considered significant indicated by an asterisk (*). (**) indicates a *p* <.005, (****) indicates *p*<0.0001. A *p*<0.05 is considered significant indicated by an hashmark (#), (####) indicates *p*<0.0001. A *p*<0.05 is considered significant indicated by a dollar sign ($).

L-GSH supplementation for 3-months resulted in a significant increase in the levels of IFN-γ in the PBMCs (**Figure 5B**), but not in plasma (**Figure 5A**). L-GSH supplementation for 3-months also caused a significant increase in TNF-α levels in the plasma (**Figure 5C**) and PBMCs (**Figure 5D**). There was also a significant increase in the levels of IL-2 in the plasma (**Figure 5E**) and PBMCs (**Figure 5F**) at 3-months post L-GSH supplementation. A significant decrease in the levels of IL-10 was also observed in the plasma samples (**Figure 5G**) at 3-months post-L-GSH supplementation. The placebo group had no statistically significant differences in the levels of IFN-γ, TNF-α, IL-2, or IL-10 after 3-month supplementation.

Overall, these results show that a 3-month clinical intervention of L-GSH supplementation in individuals with T2DM resulted in significantly increased levels of IFN-γ, TNF-α, IL-2, and decreased levels of IL-10 when compared to before L-GSH supplementation. Clinical interventional with placebo supplementation for three months did not produce any changes in these cytokines’ levels.

### Clinical Trial Findings: Intracellular Survival of BCG Within *In Vitro* Granulomas of T2DM Subjects

**Figure 6:**
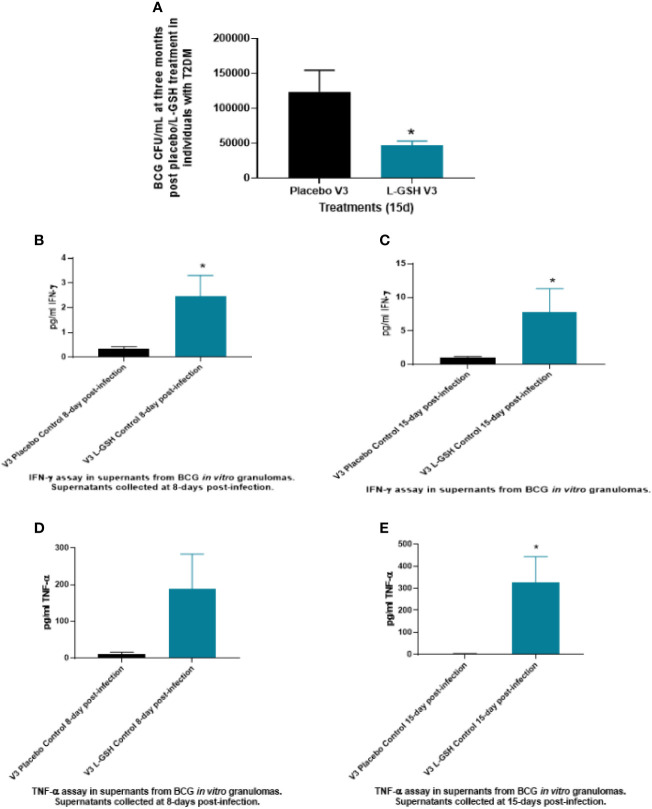
Clinical trial findings from *in vivo* and *in vitro* studies. Determination of BCG survival in the *in vitro* granulomas of T2DM subjects on L-GSH/or placebo supplement **(A)**. PBMCs isolated from individuals with type 2 diabetes at three months post- L-GSH/or placebo supplementation were infected *in vitro* with BCG and terminated at 15 days post-infection. The survival of BCG in the *in vitro* granulomas was determined by plating the granuloma lysates on 7H11. V3 represents *in vitro* granulomas generated from PBMCs isolated from subjects with T2DM at three months post- L-GSH/or placebo supplementation. ****Clinical trial findings from *in vivo* and *in vitro* studies. Quantification of IFN-γ in the *in vitro* granulomas of T2DM subjects on L-GSH/or placebo supplement **(B, C)**. PBMCs isolated from individuals with T2DM at pre- and post- L-GSH/or placebo supplementation were infected *in vitro* with BCG and terminated at 8- and 15-days post-infection. Levels of IFN-γ in the granuloma supernatants harvested at 8 days **(B)** and 15 days **(C)** were determined by ELISA using an assay kit from Invitrogen. V3 represents *in vitro* granulomas generated from PBMCs isolated from subjects with T2DM at three months post- L-GSH/or placebo supplementation. No *in vitro* treatment is denoted as the control. Clinical trial findings from *in vivo* and *in vitro* studies. Quantification of TNF-α in the *in vitro* granulomas of T2DM subjects on L-GSH/or placebo supplement **(D, E)**. PBMCs isolated from individuals with T2DM at pre- and post- L-GSH/or placebo supplementation were infected *in vitro* with BCG and terminated at 8 days and 15 days post-infection Levels of TNF-α in the granuloma supernatants harvested at 8 days **(D)** and 15 days **(E)** were determined by ELISA using an assay kit from Invitrogen. V3 represents *in vitro* granulomas generated from PBMCs isolated from subjects with T2DM at three months post- L-GSH/or placebo supplementation. No *in vitro* treatment is denoted as the control. A *p*<0.05 is considered significant indicated by an asterisk (*).

Intracellular survival of BCG was determined within *in vitro* granulomas generated from *in vitro* infected-PBMCs isolated from T2DM subjects at pre- and post- L-GSH/or placebo supplementation. When compared to the placebo group, PBMCs isolated from individuals with T2DM who received L-GSH supplementation for 3-months had a significantly reduced burden of BCG (**Figure 6A**). Overall, these results show that a 3-month clinical intervention of L-GSH supplementation in T2DM subjects significantly improved the control of BCG infection within *in vitro* granulomas generated from PBMCs of T2DM subjects when compared to the placebo group.

### Clinical Trial Findings: Quantification of Cytokines Levels Within Granuloma Supernatants of PBMCs Infected *In Vitro* With BCG in T2DM Subjects

Levels of cytokines: IL-6, IL-10, IFN-γ and TNF-α were quantified from the supernatants of *in vitro* granulomas generated from PBMCs isolated from T2DM subjects at pre- and post- L-GSH/or placebo supplementation. The PBMCs were infected *in vitro* with BCG and were terminated at 8 days and at 15 days post-infection. Only data that had significance is reported in the results and discussion.

In comparison to the placebo group, T2DM subjects post-3 months supplementation with L-GSH had a significant several-fold increase in the production of both IFN-γ (**Figures 6B, C**) and TNF-α (**Figures 6D, E**) in the granuloma supernatants from the L-GSH group at both 8-days and 15- days post *in vitro* BCG infection. The increased production of both IFN-γ and TNF-α in the L-GSH group was observed without any additional antibiotic treatment (control). Overall, the results show that when compared to a placebo, a 3-month clinical intervention of L-GSH supplementation in T2DM subjects resulted in significantly increased production of IFN-γ (**Figures 6B, C**) and TNF-α (**Figures 6D, E**) within granuloma supernatants in response to an *in vitro* BCG infection.

### Clinical Trial Findings: Quantification of Cytokines Levels Within Granuloma Supernatants of PBMCs Infected *In Vitro* With *M. tb* in T2DM Subjects

Levels of cytokines: IL-6, IL-10, IFN-γ and TNF-α were quantified from the supernatants of *in vitro* granulomas generated from PBMCs isolated from T2DM subjects at pre- and post- L-GSH/or placebo supplementation. PBMCs were infected with Erdman strain of *M. tb* and were terminated at 8 days and 15 days post-infection Only data that had significance is reported in the results and discussion.

**Figure 7:**
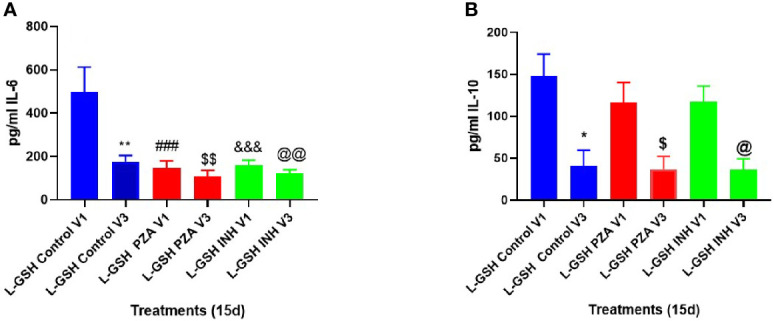
Clinical trial findings from *in vivo* and *in vitro* studies. Quantification of IL-6, and IL-10 in the *in vitro* granulomas of T2DM subjects on L-GSH supplement. PBMCs isolated from individuals with T2DM at pre- and post-L-GSH/or placebo supplementation were infected *in vitro* with *M. tb* and were either untreated (control), treated *in vitro* with PZA (5mg/ml) or INH (0.0125mg/ml) for 15 days. Levels of IL-6 **(A)**, and IL-10 **(B)** in the granuloma supernatants were determined by ELISA using an assay kit from Invitrogen. V1 represents *in vitro* granulomas generated from PBMCs from subjects with T2DM prior to L-GSH/or placebo supplementation. V3 represents *in vitro* granulomas generated from PBMCs isolated from subjects with T2DM at three months post-L-GSH supplementation. No *in vitro* treatment is denoted as the control group. A *p*<0.05 is considered significant indicated by an asterisk (*). (**) indicates a *p* <.005. (###) indicates *p*<0.0005. A *p*<0.05 is considered significant indicated by a dollar sign ($). ($$) indicates a *p* <.005. (&&&) indicates *p*<0.0005. A p<0.05 is considered significant indicated by an (@). (@@) indicates a *p* <.005.

At 3-months post- L-GSH supplementation, untreated (control) *in vitro* granulomas produced significantly less IL-6 in response to *in vitro M. tb* infection when compared to before to L-GSH supplementation (**Figures 6A**, **7**). PZA and INH treatment to *in vitro* granulomas generated from T2DM subjects at 3-months post- L-GSH supplementation also resulted in a significant decrease in the production of IL-6 when compared to before L-GSH supplementation in the untreated (control) category (**Figure 7A**).

Compared to before L-GSH supplementation, the untreated (control) category had a significant 3-fold decrease in the levels of IL-10 from the *in vitro* granulomas generated from T2DM subjects at 3-months post- L-GSH supplementation (**Figure 7B**). PZA and INH treatment to the *in vitro* granulomas generated from T2DM subjects at 3-months post- L-GSH treatment also resulted in a significant 3-fold decrease in the levels of IL-10 when compared the untreated (control) category before L-GSH supplementation (**Figure 7B**).

Overall, these results show that a 3-month clinical intervention with L-GSH supplementation in T2DM subjects resulted in significantly less production of IL-6, and IL-10 in response to an *in vitro M. tb* infection when compared to T2DM subjects prior to L-GSH supplementation.

## Discussion

In our current study we investigated the effects of a 3-month supplementation with oral L-GSH in T2DM individuals *M. tb* or *M. bovis* BCG (BCG)infection. To determine the effects of clinical intervention with L-GSH for three months on improving oxidative stress and improving mycobacterial responses against *M. tb* in individuals with T2DM, we first evaluated the levels of GSH, MDA, and various cytokines in their plasma, RBCs, and PBMCs before and after supplementation with L-GSH or placebo. We also conducted *in vitro* studies by infecting isolated PBMCs with either *M. tb* or BCG to generate *in vitro* granulomas to determine whether *in vivo* L-GSH supplementation improved immune responses against mycobacterial infection. Additionally, we evaluated the levels of various cytokines within the *in vitro* granuloma supernatants generated from the infection studies.

T2DM, a chronic metabolic disease is associated with oxidative stress from the overproduction of reactive oxygen species (ROS) and decreased efficiency of scavenger systems (Collier et al., 1990; Powell et al., 2004). Oxidative stress-induced generation of proinflammatory cytokines, including IL-6 and TNF-α, mediates ROS production, further increasing oxidative stress (Zhang et al., 2012; Donath, 2013). Previous findings from our lab also indicate that T2DM individuals with HbAlc levels above 7% consistently demonstrated a significant decrease in levels of GSH, increased levels of oxidative stress, and higher *M.tb.* burden when compared to healthy individuals without T2DM (Lagman et al., 2015). Our lab has also shown that GSH deficiency is correlated with increased levels of free radicals and intracellular *M.tb.* viability (Venketaraman et al., 2008). Therefore, we first conducted preclinical studies in healthy and T2DM individuals to confirm these previous studies findings on the differences between healthy and T2DM individuals’ immune responses against an *in vitro M. tb* or BCG infection. We observed that compared to the healthy group, the T2DM group had a significantly increased burden of both *M. tb* (**Figure 2A**) and BCG (**Figure 2B**). The significantly increased growth of intracellular *M. tb*. and BCG supports the previous findings on T2DM patients’ reduced ability to control mycobacterial infections and thus having poorer outcomes than healthy individuals (Jeon and Murray, 2008; Lagman et al., 2015; Saing et al., 2016; Ferlita et al., 2019). As T2DM patients are known to have decreased levels of GSH, not surprisingly, we also found that the T2DM group had a 4-fold reduction of GSH levels in plasma when compared to the healthy group (**Figure 3A**). Correlated with this decrease in GSH levels, we found that the T2DM group also has significantly higher levels of MDA (**Figures 3B, C**) and higher levels of the proinflammatory cytokine IL-6 (**Figure 3D**) than the healthy group. These results again are not a new discovery and only act to strengthen the existing literature and our previous findings that T2DM individuals have an overproduction of ROS and proinflammatory cytokines, while also having decreased levels of GSH.

Supplementation with L-GSH for three months in T2DM subjects overall was able to reduce oxidative stress and decrease levels of oxidized GSH (GSSG), while maintaining levels of reduced GSH (GSH) within various blood components. We measured oxidative stress levels through malondialdehyde (MDA), a naturally occurring product of lipid peroxidation, and an established marker for oxidative stress. We found that supplementation with L-GSH for three months was able to reduce the levels of MDA in all blood components, including plasma (**Figure 4C**), PBMCs (**Figure 4D**), and RBCs (**Figure 4E**). The placebo group had either an increase or no change in oxidative stress, as shown by a significant increase in MDA in RBCs (**Figure 4E**) or no change in MDA in plasma (**Figure 4C**) or PBMCs (**Figure 4D**). Furthermore, there was a significant reduction in the levels of the proinflammatory cytokine IL-6 in both the plasma (**Figure 4A**) and PBMCS (**Figure 4B**) from T2DM subjects who received L-GSH supplement. In contrast, the placebo group resulted in increased levels of IL-6 or the same levels of IL-6 in plasma (**Figure 4A**). Furthermore, supplementation with L-GSH in T2DM subjects, resulted in maintained levels of reduced GSH (**Figures 4F–H**), while the group that received placebo had decreased levels of reduced GSH after 3 months (**Figures 4F–H**). We also saw that with placebo supplementation, the levels of oxidized GSH significantly increased, while levels of reduced GSH significantly decreased in all blood components. As seen in the preclinical results, GSH was at a 4-fold reduction in theT2DM group when compared to the healthy group, therefore supplementing with L-GSH can maintain GSH levels, decrease the levels of oxidized GSH, decrease the levels of the proinflammatory cytokine IL-6 and other oxidative stress markers in individuals with diabetes.

Immunity against *M. tb* is mainly driven by Th1 cells that secrete IFN-*γ*, IL-2, and TNF-α. IL-2 is responsible for maintaining T-cell viability, and therefore promotes T-cell responses. Both IFN-γ and TNF-α are key players in the induction of cell-mediated immunity and granuloma formation to effectively control an *M. tb* infection. IFN-γ is responsible for macrophage activation, enhanced antigen presentation, and induction of nitric oxide-mediated killing mechanisms (Cavalcanti et al., 2012). TNF-α stimulates the migration of immune cells to the site of infection and is essential for the maintenance and formation of granulomas (Sedgwick et al., 1999). TNF-α also synergizes with IFN-γ to simulate the production of reactive nitrogen intermediates for mycobacterial killing within macrophages (Cavalcanti et al., 2012).

Our preclinical studies confirmed the previous findings that the T2DM group has lower levels of IFN-γ (**Figure 3E**) and TNF-α (**Figure 3F**) when compared to healthy individuals. These lowered baseline levels of IFN-γ and TNF-α may represent the cytokine dysregulation and altered immunity among individuals with T2DM that reduced their ability to control *M. tb* infection. We found that L-GSH supplementation in T2DM subjects modulated levels of IFN-γ, TNF-α, IL-2, and IL-10 within plasma and PBMCs from T2DM subjects. Post- L-GSH supplementation, we found increased levels of IFN-γ in PBMCs (**Figure 5A**), increased levels of TNF-α in plasma (**Figure 5C**) and PBMCs (**Figure 5D**), and increased levels of IL-2 in plasma (**Figure 5E**) and PBMCs (**Figure 5F**) when compared to before L-GSH supplementation. There was also a significant decrease in IL-10 in plasma (**Figure 5G**) after L-GSH supplementation. IL-10 has an immunoregulatory function and mainly acts as an immunosuppressive cytokine. IL-10 suppresses the production of IFN-γ, TNF-α, and IL-12 and also works to block actions of antigen-presenting cells (Cavalcanti et al., 2012). The observed decreased levels of IL-10 could be diminishing it’s immune suppressing effects and therefore aligns with the observed increase in IFN-γ and TNF-α. We would also like to note that the significant changes in the cytokine levels for IFN-γ and IL-10 were observed between the pre- and post-GSH groups and when the L-GSH group was compared to the placebo group. However, specifically for TNF-α and IL-2 increased levels were only significant between the pre- and post- L-GSH group, but not when the L-GSH group was compared to the placebo group. Therefore, the data supports supplementing with L-GSH was able to increase the levels of IFN-γ, and decrease the levels of IL-10 When pre- and post- placebo groups were compared, there were no significant changes in any of these cytokine levels.

The antimycobacterial effects of PZA and INH has been well established., but our lab wanted to specifically test the effects of these first-line antibiotics at 1/10 the minimum inhibitory concentration against *M. tb*. Previous published findings from our lab have showed that using *in vitro* antibiotics at 1/10 MIC PZA (5 μg/ml) and 1/10 MIC INH (0.0125 μg/ml) was able to reduce the burden of *M.tb*. in healthy individuals and we wanted to investigate this for T2DM individuals (Abrahem et al., 2019). In our preclinical studies, we provided *in vitro* first-line antibiotic treatment at 1/10 the MIC of PZA and INH to *in vitro* granulomas derived from PBMCs of T2DM individuals and measured the intracellular survival of *M. tb*. We found that compared to the untreated categories, treatment with 1/10 the MIC of PZA or 1/10 the MIC of INH resulted in a significant decrease in the burden of intracellular *M. tb* at both 8 days (**Figure 3G**) and 15 days post-infection (**Figure 3H**). Thus, demonstrating the efficacy of using 1/10 the MIC for PZA or 1/10 the MIC INH in improving the control of *M. tb* within *in vitro* granulomas of T2DM patients.

L-GSH supplementation in individuals with T2DM for 3-months also resulted in improved control of *in vitro* BCG infection when compared to the placebo group (**Figure 6A**). These results indicate that *in vivo* L-GSH supplementation in T2DM individuals could improve their ability to control a BCG infection within granulomas, compared to placebo. The improved ability to control a BCG infection for the L-GSH group, when compared to the placebo group, also correlated with significant increases in the levels of IFN-γ (**Figures 6B, C**) and TNF-α (**Figures 6D, E**) in the supernatants of *in vitro* granulomas. The increased levels of IFN-γ and TNF-α observed within granulomas supernatants along with the decreased burden of BCG could potentially be from the L-GSH supplement that modulated the cytokines for a more effective response against mycobacterial infection in individuals with T2DM.

For the *in vitro* infection studies with *M. tb*, we also compared cytokine levels before and after L-GSH supplementation within supernatants from *in vitro* granulomas. The *in vitro* granulomas were generated from PBMCs isolated from T2DM subjects, as they were in the BCG infection studies. T2DM subjects on L-GSH supplementation have significantly reduced levels of IL-6 (**Figure 7A**) in all categories, including with *in vitro* PZA and INH treatment, when compared to the control category before L-GSH supplementation. There was also a 3-fold reduction in IL-10 after L-GSH supplementation in every category when compared to the control category before L-GSH supplementation (**Figure 7B**). L-GSH supplementation overall seems to help improve mycobacterial infection control, potentially through modulating various cytokine levels to help the host combat the infection.

In conclusion, these findings demonstrate that a clinical intervention for 3-months with oral L-GSH in individuals with T2DM can decrease levels oxidized GSH, while maintaining levels of reduced GSH and overall reducing the extent of oxidative stress. In conjunction with improving redox imbalance, L-GSH supplementation was able to improve the control of *in vitro* mycobacterial infections. This improved ability to control intracellular mycobacteria could be through the improved redox homeostasis in the cell and possibly through modulation of cytokines that result in a more effective immune response against these infections. More studies should be conducted to investigate whether the observed increases in cytokines IFN-γ, TNF-α, IL-2, and decrease in IL-10 found in granuloma supernatants is indeed the cause of the decreased burden of mycobacterial. As the prevalence of T2DM increases globally and is recognized as a reemerging risk and challenge to manage TB, more research should investigate the possible benefits of using L-GSH as a potential adjunct therapy alongside current anti-TB treatments or even as a preventative strategy for individuals with T2DM with known dysregulation of GSH metabolism and increased oxidative stress.

Some of the limitations of our study are the small sample size, resulting from non-compliance and study drop-out during the rise of the global COVID-19 pandemic. Other limitations include the fact that the *in vitro* granuloma model used is not an exact replication of *in vivo* granulomas which have a more complex and dynamic environment containing other cells like fibroblasts, collagen matrix, and other white blood cells. The *in vitro* granuloma model can help us understand some factors that might translate to the more dynamic *in vivo* granuloma that humans develop in response to infections like *M. tb.* Other limitations involve our methods with PBMC lysates being used for cytokine measurements. This method has its pros and cons, while being helpful to measure cytokines potentially at a more localized level, these PBMCs can be affected by cryopreservation, length of storage, and the overall processing methods. We believe the strengths of our study is investigating the novelty of using oral L-GSH supplementation in people with T2DM for mycobacterial infections, as well as our use of the *in vitro* granuloma model that give an ideal of how *in vivo* granulomas would respond to *in vivo* oral L-GSH supplementation. We hope our findings here could lead to more studies looking into L-GSH supplementation as a possible adjunct therapy in addition to the standard normal antibiotic treatment for mycobacterial infections.

## Data Availability Statement

The original contributions presented in the study are included in the article/supplementary material. Further inquiries can be directed to the corresponding author.

## Ethics Statement

This study was approved by Western University of Health Sciences Institutional Review Board (IRB); protocol #FB17/IRB/031. All participants gave written informed consent before enrollment into the study. The patients/participants provided their written informed consent to participate in this study.

## Author Contributions

The following authors: KT, RC, AY, JO, TN, KS, CV, MS, and ET were involved in both subject recruitment and conducting the assays. AM, EA, JV, CP, and AS helped with recruitment of study subjects. This project was conceived by VV. VV was also involved in study design, obtaining institutional approvals and funding, and data analysis. All authors contributed to the article and approved the submitted version.

## Funding

Parts of this clinical trial were funded by Your Energy Systems who also provided the ReadiSorb™ LRG oral liposomal glutathione and placebo supplement for the study. We appreciate the funding support from National Heart Blood Lung Institute at the National Institutes of Health (NIH) award 1R15HL143545-01A1.

## Conflict of Interest

The authors declare that the research was conducted in the absence of any commercial or financial relationships that could be construed as a potential conflict of interest.
